# The Effect of Freeze-Thaw Conditions on Arctic Soil Bacterial Communities

**DOI:** 10.3390/biology2010356

**Published:** 2013-02-28

**Authors:** Niraj Kumar, Paul Grogan, Haiyan Chu, Casper T. Christiansen, Virginia K. Walker

**Affiliations:** 1 Department of Biology, Queen’s University, Kingston Ontario, K7L 3N6, Canada; E-Mails: niraj.kumar@queensu.ca (N.K.); groganp@queensu.ca (P.G.); hychu@issas.ac.cn (H.C.); christiansen.c@queensu.ca (C.T.C.); 2 Department of Biomedical and Molecular Sciences, School of Environmental Studies, Queen’s University, Kingston Ontario, K7L 3N6, Canada

**Keywords:** climate change, arctic soils, freeze-thaw, phylogenetic composition, fatty acids, bacteria, chaos theory

## Abstract

Climate change is already altering the landscape at high latitudes. Permafrost is thawing, the growing season is starting earlier, and, as a result, certain regions in the Arctic may be subjected to an increased incidence of freeze-thaw events. The potential release of carbon and nutrients from soil microbial cells that have been lysed by freeze-thaw transitions could have significant impacts on the overall carbon balance of arctic ecosystems, and therefore on atmospheric CO_2_ concentrations. However, the impact of repeated freezing and thawing with the consequent growth and recrystallization of ice on microbial communities is still not well understood. Soil samples from three distinct sites, representing Canadian geographical low arctic, mid-arctic and high arctic soils were collected from Daring Lake, Alexandra Fjord and Cambridge Bay sampling sites, respectively. Laboratory-based experiments subjected the soils to multiple freeze-thaw cycles for 14 days based on field observations (0 °C to −10 °C for 12 h and −10 °C to 0 °C for 12 h) and the impact on the communities was assessed by phospholipid fatty acid (PLFA) methyl ester analysis and 16S ribosomal RNA gene sequencing. Both data sets indicated differences in composition and relative abundance between the three sites, as expected. However, there was also a strong variation within the two high latitude sites in the effects of the freeze-thaw treatment on individual PLFA and 16S-based phylotypes. These site-based heterogeneities suggest that the impact of climate change on soil microbial communities may not be predictable *a priori*; minor differential susceptibilities to freeze-thaw stress could lead to a “butterfly effect” as described by chaos theory, resulting in subsequent substantive differences in microbial assemblages. This perspectives article suggests that this is an unwelcome finding since it will make future predictions for the impact of on-going climate change on soil microbial communities in arctic regions all but impossible.

## 1. Introduction

### 1.1. Will Climate Change Stress Arctic Soil Communities, and What Are the Likely Ecological Impacts?

Although low temperatures in the Arctic result in vast tracts of frozen ground or permafrost, the temperature of the soil is ameliorated by an insulating snow pack. As a result, snow depth and timing of first snow accumulation are important for the survival of subnivean life [1,2]. Prior to snow accumulation in autumn, and during the spring melt, dynamically fluctuating air temperatures are common and can result in freeze-thaw cycle (FTC) events in surface soils. Such freeze-thaw fluctuations are of ecological interest because of their possible impacts on soil microbial communities, soil carbon and nutrient transformations, as well as plant productivity [3,4,5,6,7,8]. In a changing climate, the Arctic is expected to undergo substantial warming with a projected increase in average air temperature of 4–8 °C during this century [9,10]. Although this may impact all seasons, our particular interest is in earlier spring warming, as well as the potential decrease in snow cover that together may result in more FTC incidents [11]. Climate change scenarios also predict increased variability in climate, with greater amplitude fluctuations in air temperature and precipitation, which may further enhance the frequency of soil FTCs [10,12].

Freeze-thaw events have been linked to declines in soil microbial biomass carbon [13,14,15], a proxy for microbial community size. In extreme cases, FTCs have been associated with microbial dieback of 40–60% [5,16,17]. Even a single FTC can cause the death of up to 50% of microbes [18]. Nevertheless, there is conspicuous lack of consensus among studies on the effects of FTCs on soil microbial biomass and activities [8] with other reports showing a subtle or insignificant impact (e.g., [4,6,19,20]). Some apparent inconsistencies between experiments could be attributed to the methods or the analysis, but others may depend on soil type and the severity of the experimental FTC regimes compared to naturally occurring FTCs. Both regional and landscape topographic location may be critical since they determine local climate. As a result, soils derived from sites subjected to “harsh and changing environmental conditions” would be expected to contain a relatively high abundance of indigenous FTC-resistant species [21]. In consequence, perhaps arctic soils from such locations will show little impact from any additional freeze-thaw stresses related to climate change.

Soils subjected to freeze-thaw regimes may release labile carbon and nutrients from lysed microbial cells and this has been associated with short-term peak respiratory pulses of N_2_O and CO_2_ [6,14,22,23]. A single FTC resulted in respiratory losses accounting for up to 15% of microbial biomass carbon [22]. Potentially, then, FTCs could have a significant impact on tundra carbon balance. Indeed, Schimel and colleagues [7] calculated that a freeze-thaw event could release carbon to the atmosphere corresponding to as much as 25% of the net annual primary production in an Alaskan tussock tundra region. Although release of more carbon will further exacerbate climate change, freeze-thaw induced microbial loss is crucial for arctic nutrient dynamics. Arctic vegetation growth is strongly limited by nutrient availability [24] in part due to strong microbial immobilization of soil nutrients [25]. Therefore, the release of microbial nitrogen and phosphorous from microbial cells that were lysed by FTCs could stimulate plant production and, hence, carbon uptake from the atmosphere. This would help to counteract the CO_2_ release associated with lysis-enhanced respiration. Overall, since the carbon to nutrient ratio of plants is higher than that of microbes or soil [26], FTCs could eventually contribute to a net decrease in atmospheric CO_2_ concentration. However, this prediction is critically dependent on plants being able to acquire the released nutrients at the time of microbial lysis [27]. To date, there is little evidence for this except for evergreen shrubs or perennial sedges in the arctic spring freeze-thaw period, and for graminoids in the early autumn [27,28,29]. If valid, however, FTC-mediated nutrient release could then ultimately shift plant community structure in favor of functional groups that can best capitalize on pulses of these liberated molecules. Nutrients released from FTC-lysed microbial cells that are not taken up by plants, may be acquired by surviving soil microbes, leached downslope, or lost to the atmosphere via dentrification (for nitrogen only).

Similar to FTCs, the drying and subsequent rewetting of soils may strongly affect microbes due to the rapidly changing osmotic potentials [7]. During a rewetting event, microorganisms release cytoplasmic nutrients, constituting up to 60% of the microbial biomass carbon [30,31], resulting in short-term pulses of enhanced CO_2_ release and nutrient availability (e.g., [31,32,33]). CO_2_ release and changes in microbial community composition following rewetting are usually less pronounced in soils frequently exposed to fluctuations in soil water potential *in situ* (e.g., [31,33,34]). Again, this suggests that community adaptations for stress resistance are shaped by local climate history. While drying/rewetting events have been principally addressed in relation to episodic rainfall, arctic soils are often subjected to the combination of freeze-thaw and drying-rewetting stresses in late winter [35]. Added to these stresses, in late winter, arctic soils are dried by sublimation due to the increase in sunlight, particularly in soils without much snow cover and adjacent to darker vegetation and roots, which can adsorb solar radiation [35]. As warmer air temperatures initiate above ground, snow and ice melt, with water percolating down into the frozen soil through these sublimed crevices, soil pores, frost-induced cracks, and dendritic channels [36,37,38].

### 1.2. Freeze-Thaw: Survival of the Fittest, or an Assemblage of Defenses?

Temperature changes can be challenging to microbial communities. Low temperatures and FTCs can affect protein structure and function, membrane fluidity and be associated with cellular damage due to the impact of oxidative and osmotic stresses [39,40]. Internal ice formation is largely avoided *in situ*, but the protective effect of an increase in cellular solute concentration [39] can itself result in damage, as can thawing leading to rapid changes in osmotic potential. External ice formed at low rates of cooling consists of large ice crystals [41], which are potentially harmful. During prolonged periods near 0 °C, or during freeze-thaw, ice recrystallization can result in still larger ice crystals that may contribute to further damage. Despite these many challenges, psychrophiles and psychrotolerant microbes have developed a range of physiological adaptations to survive freeze stress, allowing them to remain active at, and below, freezing conditions [42,43,44,45]. At subzero temperatures, microbial activity is controlled by the availability of unfrozen water films on soil particles [46,47], and by substrate limitations [48]. Adaptations include metabolic adjustments [49], and may involve a switch from the utilization of carbon-rich litter during thaw periods to the recycling of nitrogen-rich internal products as well as dead microbes during freeze intervals [48,50,51].

Different isolates show strikingly different susceptibilities to FTCs. For example, *Chryseobacterium* sp. C14 showed no loss of viability after 48 FTCs, resulting in a level of recovery that was three orders of magnitude higher than more vulnerable strains [52]. This species conferred some benefit to other isolates, demonstrating that experiments investigating the effect of FTCs and spring runoff should utilize assemblages, rather than individual isolates. Consortia containing cooperative species could be relatively resilient when faced with the multiple stresses associated with seasonal changes. This could partially explain the little impact seen in response to freeze-thaw stress in several studies, as well as a more marked effect in others (e.g., [6] *vs.* [15]). Whether FTCs are the cause or not, it is now fairly well established that the active microbial soil community changes seasonally, resulting in distinct summer and winter arctic [53], subarctic [54], and alpine [55,56] ecosystems. Generally, fungi dominate the tundra in winter and to a lesser degree in summer when bacterial abundance rises in the relatively warm soils [55,57]. Such seasonal assemblage shifts could reflect differential stress susceptibility or the capacity to have a vulnerability complemented by other members of the consortium. If the enhanced resilience of soil microbial communities to FTCs can indeed be attributed to adaptation to a particular local climate associated with a geographic region [21], this prompts us to consider that arctic soils from climatically distinct locations could then show substantial variation in their responses to FTCs related to climate change. It was this speculation that prompted us to undertake a small, but multi-spatial scale analysis; we report our results as part of this perspectives article in order to underscore the need for further investigation.

## 2. Experimental Section: The Effect of Simulated Freeze-Thaw Cycles on Latitudinally Distinct Soils

**Figure 1:**
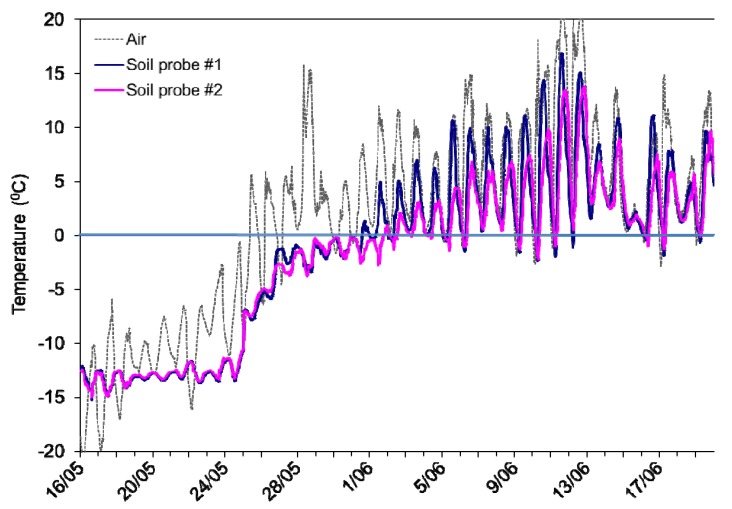
Temperatures in the air (40 cm above the ground surface) and in the soil (2 cm depth) at two locations at least 2 m apart beneath birch hummock tundra vegetation at the Daring Lake site. Temperatures were measured in the spring (2005) with dates as indicated on the X-axis. Data were collected every 30 min using copper-constantan thermocouples and a datalogger.

We hypothesized that rapid temperature changes that result in soil freeze-thaw fluctuations could alter soil microbial diversity. Evidence for multiple FTCs was apparent at a low arctic site (Figure 1) and we speculated that the FTCs seen at this geographic location could serve as a proxy for the impact of more extreme future climate change at higher latitudes. A recent analysis of climatic trends over the past ~50 years across Canada (albeit largely but not entirely based on data from relatively southerly weather stations) indicates that the frequency of soil FTCs is generally higher at sites with relatively warm mean annual air temperatures (*i.e.*, at lower latitudes), and especially in relatively warm and dry winters [58]. Furthermore, these data suggest that climate change will increase the frequency of soil FTCs at most sites over the next 50 years [58]. Accordingly, we sampled replicate soils from three distinct sites in the Canadian low, mid- and high Arctic and used spring air and soil temperature data collected *in situ* at the low arctic location as the basis for FTC treatment of soils from all three sites. As indicated, we present our perspective on the effect of freeze-thaw events on soil microbes, and show the results of our biochemical analyses on the impact of freeze-thaw events on these three geographically distant assemblages.

### 2.1. Soil Collection Sites, Freeze-Thaw Regime and Respiration Monitoring

Soil samples were collected in triplicate from three different sites in the Canadian Arctic: Daring Lake, Cambridge Bay, and Alexandra Fjord representing the low (64°52' N 111°35' W), mid- (69°11' N 104°45' W), and high Arctic (78°53' N 75°47' W), respectively. Some soil biochemical and climatic variables for these sites are listed in Table 1. At each site, soils were obtained from three separate but similar locations (20–100 m apart) close to the top of exposed ridges where soil freeze-thaw fluctuations are most likely because of thin or absent snowcover. Samples of the top 2–3 cm of the soil organic layer were taken in spring (Daring Lake) and summer (the higher latitude sites) and shipped to our lab within several days and stored at −20 °C until processed [59]. Ideally, soils would be subjected to FTCs immediately, but climate-prescribed seasonal differences in the collection dates, transport availability to remote sites, and the requirement to randomize the microcosms in the experimental apparatus dictated otherwise. The Daring Lake site was on top of a wind-exposed upland esker consisting of dry heath soils with a dominant vegetation of lichens *Cladina* sp*.*, and dwarf shrubs, *Ledum decumbens*, *Betula glandulosa*, and *Empetrum nigrum*. Shrub cover at the Cambridge Bay site was dominated by *Dryas integrifolia*, with some sedges *Carex* sp., willows *Salix* sp*.* and mosses. The Alexandra Fjord soils were collected from a high arctic oasis with vegetation mainly consisting of sedges *Eriophorum* sp*.*,* Carex* sp*.* and arctic willow, *Salix arctica*.

**Table 1:** Comparison of some soil biochemical and climatic variables including pH, organic layer (org layer) depth, soil carbon and nitrogen, mean annual temperature (AT), mean growing season temperature (GST), average annual precipitation (AP), snow depth, and average number of days above 0 °C for the three sites, Daring Lake (DL), Cambridge Bay (CB) and Alexandra Fjord (AF).

Site	pH	Org layer (cm)	Soil C (%)	Soil N (%)	Soil P (ppm)	AT (°C)	GST (°C)	AP (mm)	Snow depth (cm)	Days above 0 °C
DL ^1^	4.3	2–5	20	0.7	21	–9	3	150	29	127
CB ^2^	6.6	5–6	24	1.4	7	–14	6	140	31	79
AF ^3^	5.6	NA	12	0.8	4	–15	10	250	NA	~65

^1^ data from this study and Chu *et al*., 2010 [60], with climatic data from Bob Reid, Department of Indian and Northern Affairs, Water Resources Division, NWT; note that the days above 0 °C is reported as the diel average temperature above 0 °C. ^2^ data from Chu *et al*., 2010 [60], with climatic data from the National Climate Data and Information Archive, Environment Canada (1971–2000); note that the days above 0 °C is reported as the minimum diel temperature above 0 °C. ^3^ data from Chu *et al*., 2010 [60], with climatic data averaged from Labine, 1994 and Rayback and Henry, 2006 [61,62], with days above 0 °C reported as “frost-free days” for Quttinirpaaq National Park, Ellesmere Island (Environment Canada); NA, indicates that the information is not currently available.

After thawing, obvious roots and stones were quickly removed by hand prior to biochemical analysis. Triplicates were composited, packed in sealed polypropylene containers (100 g soil in each of three 218 mL containers for each site), and subjected to multiple freeze-thaw temperature fluctuations. In order to use temperatures that were consistent with the low arctic site, we examined temperature records of the soil (2 cm depth into the soil organic layer) and air temperatures (40 cm above the ground surface) from the Daring Lake weather station (Figure 1). Since the exposed sites did not have the benefit of insulating snow cover, we made the assumption that the temperature of the top centimeters of soil would be the same as the air temperature. Indeed, records show that the likelihood of freeze-thaw diminishes greatly with depth from the soil surface down into the deeper soil horizons, and that soil FTCs may occur in either spring or fall. Climate change may eventually shift climates northwards so that the mid- and high arctic sites experience conditions more similar to current temperature patterns at the low arctic site [55]. Therefore, we exposed soil from all three sites to a 14-day treatment period of the same temperature regime. This approximated the air temperatures just above the snow surface observed at the low-arctic site in the 2005 field season (*i.e*., oscillating from −10 °C to 0 °C and back once per day; Figure 1). We connected the soil containers to a CO_2_ detector, a gas switcher and a computer for data acquisition (Qubit Systems, Kingston, Ontario, Canada) in order to monitor microcosm respiration throughout the incubation period.

### 2.2. Soil Phospholipid Fatty Acid and DNA Analyses

Each of the triplicate soil samples from each site was subjected to phospholipid fatty acid (PLFA) analysis. Fatty acid methyl esters were extracted as described using the Microbial Identification System (Microbial ID Inc. [MIDI], Newark, DE, USA) as previously cited [63,64]. Briefly, soil samples (3 g) were saponified (100 °C in 3 mL 3.75 M NaOH in 50% methanol, 30 min), methylated (80 8C in 6 mL of 6 M HCl in 54% for 10 min), extracted (in 3 mL of a mix of equal volumes of methyl-tert-butyl ether/hexane for 10 min), and washed (1.2% NaOH for 5 min). This procedure and the gas chromatography of the resulting esters were conducted by Keystone Labs, Edmonton, Alberta. Only those fatty acids that were the most abundant (>1% of chromatographic peak areas for either control or treated samples) were considered for analysis. The total average peak area for the triplicate samples and controls were converted to ratios of PFLA peak areas in the experimental series over their corresponding controls to facilitate comparisons between untreated and treated soils. This was done because the focus of these experiments was not to describe the fatty acid composition at each site in detail but determine if FTCs would perturb PLFA profiles.

DNA was extracted using a soil isolation kit (PowerSoil™ DNA Isolation Kit, MO BIO Laboratories, Inc., Carlsbad, CA, USA) as per the manufacturer’s instructions. Polymerase chain reaction denaturing gradient gel electrophoresis (PCR-DGGE) was conducted as previously described [65] except that the DNA was not treated with ethidium monoazide. PCR-DGGE was performed three or more times on each sample to ensure reproducibility of the gel patterns.

For pyrosequencing, DNA was extracted from all 18 soil samples (controls and FTC-treated for each of the three geographic regions). The DNA samples were quantified using a Nanodrop spectrophotometer (NanoDrop-1000; Ver. 3.7.1). After multiple initial PCR and agarose gel analyses [65], all subsequent procedures were performed at the Research and Testing Laboratory (RTL: Lubbock, TX, USA) with tag-encoded FLX amplicon pyrosequencing (TEFAP) performed in accordance with established protocols [66,67]. Bacterial primers Gray28F (5'-TTTGATCNTGGCTCAG-3') and Gray519r (5'-GTNTTACNGCGGCKGCTG-3') were used to amplify ∼500 bp fragments spanning the V1 to V3 hypervariable regions of the bacterial 16S ribosomal RNA (rRNA) genes. Initial generation of the sequencing library used a one-step PCR with a total of 30 cycles, a mixture of Hot Start and HotStar high fidelity Taq polymerases, and amplicons originating and extending from the 28F primer. Analysis utilized a Roche 454 FLX instrument with Titanium reagents based on RTL protocols [68]. After sequencing, all failed sequence reads, low quality sequence ends and tags and primers were removed, with non-bacterial rRNA gene sequences and chimeras removed using B2C2 [69] as previously described [70]. To identify the bacteria in the remaining sequences, sequences were denoised, assembled into clusters and compared with 16S bacterial sequences curated at the National Center for Biotechnology Information (NCBI) using a distributed MegaBLAST .NET algorithm [71]. Using RDP ver 9 [72] to determine quality and the .NET and C# analysis pipeline, the MegaBLAST outputs were compiled, validated and further analyzed as previously described [70]. These were subsequently used for sequence identity (percent of total length query sequence aligned with a given database sequence) and validated using taxonomic distance methods, and classified at the appropriate taxonomic levels based upon standard conventions. Specifically, 16S rRNA gene sequences with 90% or more base pair identity with existing sequences in the database were resolved at the family level. Similarly, those sequences with scores between 85–90% were resolved at the order level, 80–85% at the class level and 77%–80% at the phylum level [70]. 

### 2.3. Community Responses of Freeze-Thaw Stresses in Microcosms: Results

When subjected to the alternating temperature cycles, probes inserted into the microcosm cores showed that temperatures of −10 °C and 0 °C were achieved, and ice crystals or water vapor were visible on the outer surface of the microcosms in the appropriate distinct cycling periods. There were no overall differences between the CO_2_ levels derived from the soil microcosms originating from the different geographic regions and therefore these nine profiles are not shown here. Nevertheless, respiration monitoring was consistent with FTCs, showing alternating periods of no detectible CO_2_ discharge followed by a modest burst of CO_2_ as the soil thawed (not shown). This could reflect either physical release of trapped gas, carbon mineralization of solutes from microbes that were lysed by the freeze-thaw treatment, or simply more favorable temperatures (at thaw) for microbial activity. However, these alternatives were not investigated for this study. After the two-week incubation period, there were again no significant differences in the total cumulative respiration between microcosms or in respiration rates compared to the beginning of the experiment.

**Figure 2:**
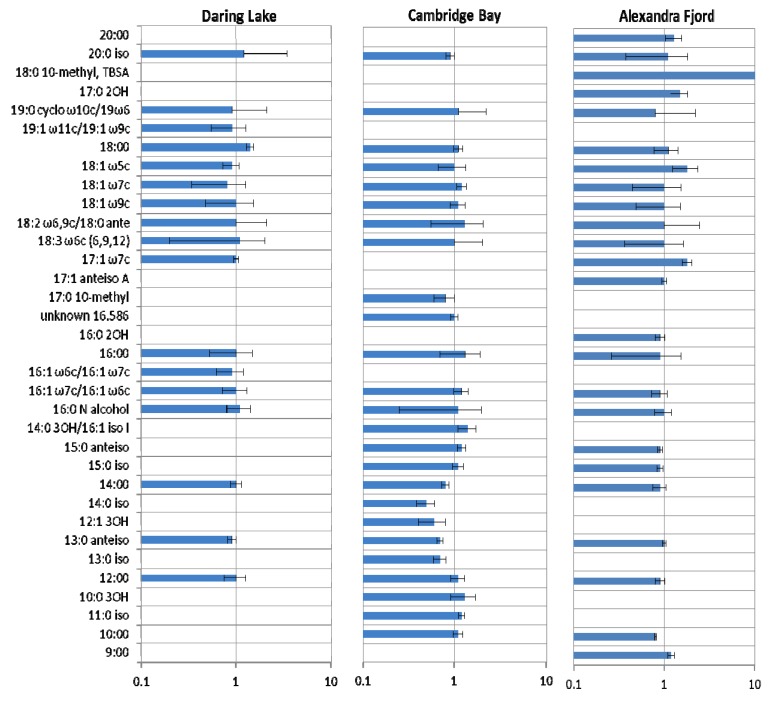
Mean ratios of the most abundant fatty acids (>1% of chromatographic peak areas) from low arctic (Daring Lake), mid arctic (Cambridge Bay) and high arctic (Alexandra Fjord) soils, both before and after daily freeze-thaw treatments for 14 days. Freeze-thaw-treated (n = 3) /untreated (n = 3) are represented along with standard errors (lines on the bars), with no change in mean abundance after treatment indicated by 1. Increases and decreases relative to mean control values are shown as bars with mean values >1 or <1, respectively. Fatty acids are named according to standard nomenclature but abbreviated where appropriate and represent from bottom to top as: 9:00, 10:00, 11:0 iso, 10:0 3OH, 12:00, 13:0 iso, 13:0 anteiso, 12:1 3OH, 14:0 iso, 14:00, 15:0 iso, 15:0 anteiso, 14:0 3OH/16:1 iso I, 16:0 N alcohol, 16:1 w7c/16:1 w6c, 16:1 w6c/16:1 w7c, 16:00, 16:0 2OH, unknown 16.586, 17:0 10-methyl, 17:1 anteiso A, 17:1 w7c, 18:3 w6c (6,9,12), 18:2 w6 9c/18:0 ante, 18:1 w9c, 18:1 w7c, 18:1 w5c, 18:00, 19:1 w11c/19:1 w9c, 19:0 cyclo w10c/19w6, 17:0 2OH, 18:0 10-methyl TBSA, 20:0 iso, 20:00 (the value for 18:0 10-methyl TBSA in Alexandra Fjord soil was 15.8 with a standard error of 1.62).

The impact of FTCs on the soil community profiles was further examined using three additional culture-independent methods: fatty acid analysis, PCR-DGGE and 16S rRNA gene sequencing. Although downstream PLFA analysis may not be recommended in experiments that subject soils to temperature variations that can result in changes to membrane lipids [73], each of the experimental microcosms was treated in the same way. Using relative FA abundance gives a measure of the FTC-mediated impact rather than an assessment of the community profile *per se*. As expected, fatty acid profiles in untreated soils differed in richness and composition among sites. For example, the Daring Lake soils contained 17 different signatures with relative abundance ≥1%, while the Cambridge Bay and Alexandra Fjord soils had 25 and 24 signatures, respectively (Figure 2). FTC-treatment had a differential effect on the soils, depending upon their origin and the particular fatty acid. Overall, fungal-associated fatty acids (e.g., 18:2 ω6,9c and 18:1 ω9c; but note that 18:1 ω9c may be an unreliable fungal signature since it is found in certain Solirubrobacterales [74,75]), did not appear to show dramatic changes in response to FTCs, as suggested by observations in previous studies indicating that fungi tend to dominate winter soils [55,57]. Overall, however, since PLFA composition varied among the sites it was difficult to determine additional general trends. In Alexandra Fjord soil samples, for example, a fatty acid indicative of the Gram-positive Actinobacteria of the Order Actinomycetales (18:0 10-methyl, tuberculostearic acid) increased 15.8-fold after freeze-thaw treatment. Indeed, the ratio of the abundance of individual fatty acids before and after the freeze-thaw treatment most readily showed the overall response patterns. The Daring Lake soils appeared relatively resilient to FTCs since only 18% (3/17) of the most abundant fatty acids showed >10% increase or decrease in the abundance ratio. In contrast, the FTC-mediated impact on PLFAs was much greater for the more northerly sites with substantive changes in 56% (14/25) and 33% (8/24) of the fatty acids derived from Cambridge Bay and Alexandra Fjord soils, respectively. Furthermore, the range of ratio changes (*i.e.*, the magnitude of increase or decrease in response to the FTC treatment) varied least among the Daring Lake soils (0.8–1.4) and most among the more northerly sites (Cambridge Bay: 0.5–1.4 and Alexandra Fjord: 0.8–1.8, excepting one signature that increased almost 16-fold).

Although each of the microcosms containing soil derived from the same site had identical banding patterns using PCR-DGGE analysis of the 16S ribosomal RNA genes, and were distinct from the banding patterns obtained from different sites, there were no clear, regular differences in band patterns after FTCs (not shown). Unlike the dramatic changes in DGGE community profiles that are evident after more stressful treatments (e.g., nanoparticle exposure, [65] and ultraviolet radiation [76]), our results initially suggested, similar to others [6], that FTCs did not appear to radically shift bacterial community structure in a predictable, consistent way.

**Figure 3:**
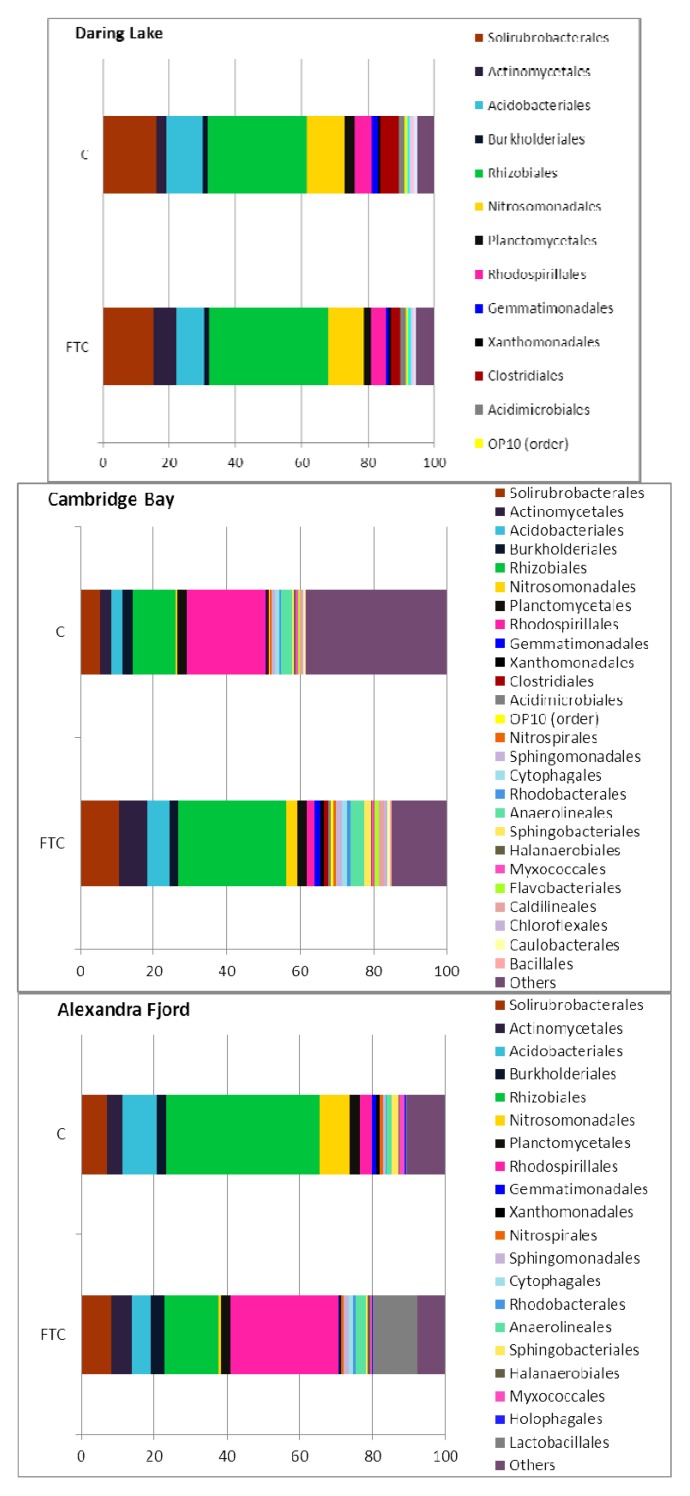
Bacterial phylogenetic composition within the soil assemblages of Daring Lake, Cambridge Bay and Alexandra Fjord, both before (control; C), and after multiple freeze-thaw cycles (FTC). Sequence identity was established after pyrosequencing of the 16S rRNA genes, classified into Orders, and the means of those with ≥1% abundance (for either treatment or control groups) presented as discrete categories, with groupings of less abundant Orders shown as “Others.”

Due to the challenge in interpreting the modest and seemingly inconsistent changes we observed in the electropherograms, 16S rRNA gene sequencing was undertaken so that any differences in the relative abundance of specific community members could be better quantified. As has been documented in other studies, DNA sequence analysis is a sensitive technique (e.g., [59,77]). Because each sample was limited to a survey of 3,000 bacterial sequence reads, we focused on bacterial phylogenetic community structure at the Order taxonomic level, and found clear differences among sites both in richness (number of Orders) and evenness (relative abundances of the Orders). For example, the Daring Lake soils contained bacteria that were classified using a cut-off level of an abundance ≥1%, into 16 different Orders, while the Cambridge Bay and Alexandra Fjord bacteria were grouped into 26 and 20 Orders, respectively (Figure 3). Solirubrobacterales, Rhizobiales, Nitrosomonadales and Acidobacteriales dominated the Daring Lake community. At the higher latitudes, Rhizobiales also dominated along with Rhodospirilalles (Cambridge Bay) and Actinomycetales (Alexandra Fjord). Soil bacterial community structure at sites similar to ours across the Arctic appears to be strongly influenced by soil pH [59]. Since soil pH varied across the sites investigated here (4.3, 5.6 and 6.6), our results suggest that even at the Order taxonomic level, pH may have a strong influence on tundra soil bacterial community structure.

**Figure 4:**
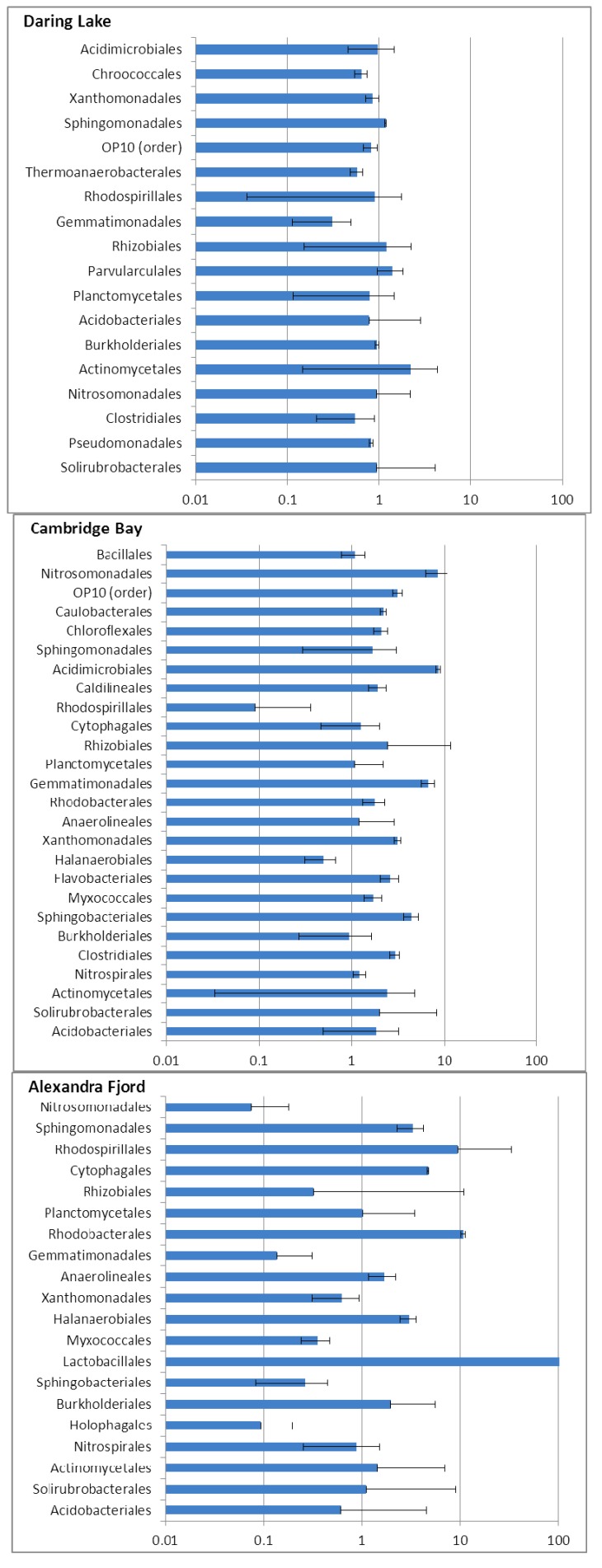
The effect of the freeze-thaw treatment on the relative abundance of each of the bacterial Orders (with those with ≥1% abundance for either treatment or control groups shown) in the soils from Daring Lake, Cambridge Bay and Alexandra Fjord. Data are ratios of the mean abundance before and after freeze-thaw treatment, with no change in the mean abundance in the Order after treatment indicated by 1. Increases and decreases relative to control values are shown as bars with mean values >1 or <1, respectively. Standard errors of the means are shown as lines on the bars. Sequences were obtained in triplicate for all controls and treatment samples. However, caution must be used in examining the FTC-treated Alexandra Fjord microcosms since one of the isolated DNA samples could not be optimally pyrosequenced; only operational taxonomic units representing the most abundant orders were reported in one of the three replicates and, therefore, this particular file set was not considered in the analysis.

Perhaps most surprisingly, the effect of FTCs on the phylogenetic composition differed depending on the originating site (Figure 3, Figure 4). After freeze-thaw treatment, the overall abundance of the major Orders from the Daring Lake site did not appear to change. This was apparent even when the data was mined for abundance at the Family level (not shown). Similarly, the same proportion of “minor” Orders (<1%) was found both prior to and after FTCs in the low arctic microcosms. In striking contrast, profiles from the higher latitude soils appeared to be more perturbed by FTCs. In particular, Cambridge Bay soils initially showed a very high level of diversity with “minor” Orders making up over 38% of the mean phylogenetic profile (Figure 3). However, this level of diversity was reduced to less than 15% after freeze-thaw treatments. Alexandra Fjord soils were not as diverse, with minor groups making up 10% of the profile, which was reduced to 7% after FTCs. In the higher latitude soils the abundant Orders also showed an impact by FTCs, with contrasting patterns in the direction and magnitude of abundance changes in response to the treatment (Figure 3, Figure 4). For example, in the Cambridge Bay soils the Nitrosomonadales, Acidomicrobiales, Actinomycetales, Gemmatimonadales, Sphingobacteriales, Rhizobiales, and Solirubrobacterales were all increased at least twofold as a result of the freeze-thaw treatment, while the Rhodospirilalles and the Halanaerobiales were reduced by at least a factor of two. By contrast, in the Alexandra Fjord freeze-thaw-treated microcosms, the mean relative abundance of Rhodospirilalles increased approximately 10-fold. The Gram-positive Lactobacillales appeared only after FTCs in the Alexandra Fjord soil (Figure 3, Figure 4). Thus, changes in the relative proportions of Orders were clearly seen in the higher latitude samples, but perhaps just as significant, the sample variation, as shown by the error bars was striking (Figure 4).Therefore, there seems to be a clear distinction on the impact of FTCs in the higher latitude soils compared to the Daring Lake site.

## 3. Discussion and Conclusions

### 3.1. Implications of the Microcosm Investigations

The frequency of FTCs in arctic environments is predicted to increase as a consequence of climate change [10,12]. Recordings of soil temperature fluctuations in late winter-early spring and in the autumn at our low arctic site showed multiple freeze-thaw events (Figure 1), which may well foreshadow future conditions at higher latitudes. Thus, we used these temperature fluctuations to design freeze-thaw stress conditions for soils collected at three latitudinally distinct sites, ranging from the Canadian low to high arctic. Under FTC conditions and at temperatures fluctuating around 0 °C, ice recrystallization can be very damaging to cell membranes [44], and this, coupled with the osmotic stress from solute concentration changes due to ice and snowmelt, may ultimately result in cell death [34]. Although, as previously indicated, some psychrophiles and psychrotolerant species contain antifreeze proteins and osmoprotectants that shield both themselves and others in the consortium from such stresses [39,52,78,79,80,81,82], many microorganisms die despite these adaptations [35,56].

PCR-DGGE profiles showed some modest changes in banding pattern, but it was not until the effects of FTC were examined in the communities by pyrosequence and fatty acid analysis that the differential impact on soils from the different sites was apparent. The bacterial assemblage from the low arctic Daring Lake site was relatively unperturbed by FTCs, as clearly evident from the phylogenetic composition. Here, we observed no change in the relative abundance of any of the major soil bacterial Orders or Families after FTC treatment and no obvious loss of diversity. This suggests a strong microbial community resilience (at these taxonomic levels) to the imposed environmental stress. Soils were deliberately sampled on relatively exposed ridges where FTCs are likely because of absent or very limited snowcover and full exposure to dynamic air temperature fluctuations. As well, because the Daring Lake site is situated at a lower latitude, it could be expected to undergo more FTCs than the more northerly sites [55]. Since the freeze-thaw experimental regime was based on temperature fluctuations measured in late winter and spring at this general location (Figure 1), it is perhaps not surprising [21] that indigenous soil microbes were relatively well adapted to survive these conditions. Similarly, hardy Antarctic bacterial communities have been reported to be relatively unresponsive across a range of FTC treatments varying in intensity [83]. In effect, the Daring Lake site, like other arctic and Antarctic environments that frequently experience freeze-thaw challenges, would be expected to be inhabited by stress-tolerant bacteria [21,83].

Contrary to the FTC-tolerant microbial assemblage from the low arctic site, mid- and high arctic soil communities appeared to be more affected by FTCs. In these higher latitude soils many of the major fatty acids showed a marked change; 56% and 33% of the fatty acids from the Cambridge Bay and Alexandra Fjord sites, respectively, showed more than a 10% change in abundance after freeze-thaw treatment, compared to 18% for the Daring Lake community. Pyrosequencing analysis revealed a similar trend with changes in the overall abundance of the bacterial Orders present prior to FTC treatment. The Alexandra Fjord soils were impacted by FTCs as evidenced by a decline in overall bacterial diversity in the minor Orders (10% to 7%), coupled with notable shifts in phylogenetic diversity. For example, after freeze-thaw treatments, there was an increased relative abundance of Lactobaccillales, Rhodospiralles, and Rhodobacterales, all of which have been reported from the Antarctic, or high arctic ice shelves and soils [84,85,86], suggesting that species within these groups may have low temperature adaptations. Nevertheless, the range of abundance values varied between microcosm replicates, thereby indicating that there was a lack of consistency in the magnitude of these increases. It is possible, then, that not only methods and analysis, but also a variable biological response, may underlie the apparent discrepancies between previously published studies [8,13,14,15,16,17,18,19,20,21] on the impact of FTCs.

For the mid-arctic site, the most notable shift in community structure was the dramatic decrease in the diversity contributed by minor Orders; this dropped more than half (38% to 15%) after FTCs (Figure 3), with some major Orders then appearing to make up more of the phylogenic composition. This striking reduction in diversity is similar to the results of FTC selection on cultured enrichments, which ultimately resulted in the recovery of microbes that were characterized by the production of osmolytes and inhibitors of ice recrystallization [52,82]. As significant as the reduction of diversity, however, was the high variation seen in the three replicate microcosms for this site compared to the relatively small variation in the Daring Lake soils (Figure 4). To illustrate, the FTC-mediated decrease in abundance of Rhodospiralles and Halanaerobiales in Cambridge Bay soils was affiliated with error bars spanning almost an order of magnitude. Thus, species within these Orders are likely susceptible to FTC-mediated conditions, but the magnitude of this vulnerability could have been mitigated in a particular microcosm by the concurrent freeze-thaw selection of a chance group of beneficial, commensal species. These latter species may be part of the Orders present in low abundance. In this regard, it would be of interest to increase the number of sequence reads so that minor groups could be more accurately enumerated, as well as to replicate each microcosm a dozen or more times to determine if any patterns in this apparent unpredictability emerge. Overall, however, the observed variation between microcosms derived from the high latitude soils strongly suggests that FTC effects on microbial community composition and relative abundance may not be absolutely predictable and that stochastic influences may play a significant role in the outcome.

Taken together, we speculate that soil microbes collected at the mid and high arctic sites were not strongly selected *in situ* for community adaptations to FTCs, compared to the Daring Lake site. Perhaps because of differences in local climate, whereby the more northerly sites typically experience fewer FTCs [55], both the higher latitude sites were noticeably impacted by freeze-thaw treatments. In particular, Cambridge Bay microcosms showed an overall reduction in diversity. The question then becomes: to what extent do disturbed consortia function differently compared to the original community? Bacteria are some of the most abundant and diverse organisms on earth [87], and our knowledge of community structure and its linkages to microbial biogeochemical activity is severely limited. For instance, studies on the temperature response of arctic and Antarctic microbial communities report not only variations in bacterial species, but highly divergent changes in the gene expression of the major functional genes such as nifH, nosZ and amoA involved in the soil nitrogen cycle [83,86,88,89]. In this case then, it is very difficult to predict directional effects of climate warming on nitrogen cycling by the community as a whole, and likewise, more generally, for all other critical biogeochemical processes. Furthermore, changes in microbial composition may not affect ecosystem process rates if the post-disturbed community (with a different composition/structure) contains taxa that are functionally equivalent to those that were previously abundant in the pre-disturbed community [90]. Likewise, new taxa in the post-disturbed community may function differently, but still maintain pre-disturbance community process rates. In summary, the inherent genetic variability and potential for rapid acclimation and adaptation in soil bacterial communities undoubtedly plays an important role in retaining a full range of ecosystem functions even after such environmental stress.

### 3.2. Implications for Predictions on the Effect of Climate Change

It is undoubtedly a challenge to predict directional shifts in arctic bacterial communities in response to climate change. However, what makes this difficult task virtually impossible is the evidence of stochastic variation that we have reported here. Since different microbes can employ very different tactics to evade or mitigate the impact of FTCs, including the synthesis of specialized proteins, the maintenance of a fluid membrane, the production of osmolytes and even commensal or mutualistic relationships, to mention just a few, it is perhaps no wonder that every microcosm which was exposed to novel or at least unusual freeze-thaw treatments resulted in a different community structure. We initially hypothesized that soils from higher latitudinal sites might be especially vulnerable to soil temperature changes recorded at the low arctic site, as a proxy to predict the effect of future climate change. Even if one argues that the climatic histories with respect to FTCs for each site were not that different, the fundamental point is that our data clearly show strong differences in the variability of freeze-thaw responses among sites. We now speculate that we might not have needed to collect samples at such vast distances. A similar variable response may well have been obtained using soils collected from different topographical locations at a single site if those locations varied in FTC history as a selective force on the bacterial community. We further hypothesize that if indeed the soil communities at the Daring Lake site had been “pre-selected” for relatively high numbers and amplitudes of FTCs over countless seasons, then our results together provide an explanatory mechanism for the pattern of responses.

We propose that freeze-thaw fluctuations are not near-catastrophic stresses, but can exert a sufficiently strong selective pressure to allow resource distribution and community adaptation. Adaptation in our microcosms, which harbored communities approximating 10^10^ individuals [91] and numbering more than 10^4^ different species is perhaps, in retrospect, not surprisingly a product of stochastic, chaotic mechanisms. A “butterfly effect,” as defined by chaos theory [92], results when slight initial disturbances can give rise to larger differences in a subsequent state. In our case, we posit that minor perturbations in our microcosm communities gave rise to unique species assemblages in each “non-adapted” microcosm derived from the higher latitude soils. The consequence of these observations is rather sobering; regretfully, we must submit that the effect of climate change on arctic soils may be inherently unpredictable.
